# A Sarcomatous Tumor, Containing Hair and Stearine, Removed from the Womb

**Published:** 1849-09

**Authors:** Gunning S. Bedford

**Affiliations:** Professor of Midwifery, and Diseases of Women and Children, in the University of New York


### OBSTETRICS.

*A Sarcomatous Tumor, containing Hair amd Stearine, removed from the Womb.*—By GUNNING S. BEDFORD, M. D., Professor of Midwifery, and Diseases of Women and Children, in the University of New York. —On Wednesday the 7th of April, 1847, Mr. D. called at my office, at 4 o'clock, P. M., and requested me to pay a professional visit to his wife. She had been attended for seven weeks by two professional gentlemen who, on the Sunday before I saw her, had voluntarily withdrawn their attendance, under the conviction that her case was beyond remedy, and with the opinion fully expressed to Mr. D. and his friends, that, in all probability, she would survive but a few hours. The husband, in his interview with me, spoke kindly of the physicians, and remarked that he was without the slightest hope, he and his friends having watched with the suffering patient the two previous nights, expecting her death at any moment. With such a representation of the case, I frankly told the husband I thought a visit from me useless, but if it would afford him any gratification I would cheerfully accompany him. He repeated his desire that I should see his wife; and, on being introduced into her chamber I found her lying on her back, her face pale and emaciated, with every indication of excessive prostration; the expression of her countenance, too, gave evidence of great suffering. Her pulse was thready, and beat 120 to the minute. Such was her exhaustion, that, when I addressed a question to her, it became necessary for me to place my ear close to her lips to distinguish her answer, and then her articulation was almost inaudible. In fact the appearance of the patient was that of a dying woman. Her respiration was labored, and the abdomen as much distended as is usual at the end of the ninth month of gestation. On percussing the abdomen I distinctly recognised fluctuation ; and in attempting to introduce my finger into the vagina, with a view, if possible, of ascertaining the character of the enlargement, I felt at the opening of the vulva a soft, elastic tumor projecting through the mouth of the womb, which was dilated to the size of a dollar piece. The parietes of the mouth of the womb thus dilated were extremely attenuated, and did not appear to be thicker than ordinary writing paper. I found no difficulty in introducing my finger between the tumor and internal surface of the cervix, the adhesion being so delicate as to yield to the slightest effort. I satisfied myself that there was no action in the womb ; the patient had not experienced anything like labor pains, and the dilatation of the cervix was the result merely of mechanical pressure produced by the tumor within the uterus. Whilst pressing gently with my finger on the tumor as it presented at the mouth of the womb, and grasping with the other hand the abdominal enlargement, I could again distinctly feel fluctuation, and found also that I distinctly comprehended the tumor between my two hands thus applied. Again, in placing my finger on the outer portion of the posterior lip of the uterus, and seizing with the other hand the upper surface of the tumor through the abdominal walls, alternately elevating and depressing the two hands, it was evident that I embraced the womb itself, which was immensely distended by the growth of the tumor. In making an examination *per rectum*, I could without difficulty detect the enlarged uterus. These circumstances, together with the important fact that the abdominal enlargement was uniform on its surface, possessing nothing of the features usually attending extra-uterine growths, such as ovarian and fibrous tumors, etc., caused me to arrive at the conclusion that, in the present case, the tumor was exclusively *intra-uterine*. It will be perceived that on this decision depended the remote hope of giving to my suffering and almost dying patient, even temporary relief from her agony. Having, therefore, formed my opinion as to the seat of the tumor, and partially as to its nature, I stated to the husband, that, desperate as the case was, and imminently perilous as would of necessity be any attempt to remove the tumor in the exhausted and almost hopeless situation of his wife, yet it was my opinion that the tumor could be removed—although the *serious hazard was that she would sink under the operation.* This opinion was given emphatically, without reserve, and unaccompanied by a word of comment calculated to urge consent to an operation, which presented but little prospect of permanent relief, and could only be justified by the reasonable expectation, that if the patient should survive the removal of the tumor, her suffering would be mitigated, and her progress to the grave rendered comparatively comfortable. The opinion was communicated to the patient by her husband, and she expressed an ardent desire that the operation should be performed without delay, remarking that she was prepared to encounter every thing, even death itself, with the remote hope of temporary relief from the agony occasioned by the pressure of the tumor. The husband and friends acquiescing fully in this appeal of the suffering patient, I left the house, promising to return in half an hour and perform the operation. On my return I was accompanied by my friend, Dr. Detmold, and two of my pupils, Messrs. Burgess and Woodcock.

These gentlemen heard with me the following particulars of the case as related by the husband and sister of the patient: Mrs. D. was 47 years of age, and married in 1832. Soon after her marriage she was attacked with cholera; and during her convalescence from this disease, she miscarried. Her health had been more or less infirm for the last ten years. Her menstrual periods had always been regular, with the exception last year, during which time they occurred about once in two or three months, and then not freely. This she imputed to *change of life*, and the circumstance did not attract any particular attention. Her abdomen had begun to enlarge in July, 1846, and continued to do so unto the present time. In January last, she suffered greatly from distention of the bladder, and could not void her urine except in small quantities at a time, accompanied by excessive pain. For this she consulted a medical man, who found it necessary to introduce the catheter, from time to time, to relieve the bladder. She commenced as early as January to be constipated, and defecation was attended with excruciating suffering. These difficulties about the bladder and bowels continued to increase, and for weeks before I saw her, she repeatedly passed over ten days without an evacuation—medicines having no effect, and injections per rectum immediately returning, without bringing away any fæcal matter. Her urine was voided in very small quantities, not more than two table-spoonfuls at a time, and it was nearly the color of blood. It was impossible for her to evacuate the bladder except when resting upon her elbows and knees; this position, however, occasioned so much fatigue, that, in her present exhausted condition, she could not avail herself of it. In a word, the agony of this unhappy sufferer was induced almost entirely by the pain consequent upon the attempt to evacuate either the bladder or rectum. With these facts before me, together with a knowledge of the position and bearings of the tumor, it was not difficult to arrive at the important conclusion that the pain and distress in the bladder and rectum were due to *mechanical pressure of the intra-uterine growth.* At my request, Dr. Detmold examined the patient; and, in view of all the circumstances of the case, concurred with me in opinion that *without an operation, she could survive but a few hours; whilst, if she did not sink under the attempt to remove the tumor, her distress would be sensibly palliated, and her life possibly prolonged.*

With the understanding, therefore, of the uncertainty and immediate danger of the operation—an understanding fully appreciated by the patient and her friends—I proceeded to remove the tumor in the following manner:—A mattress was arranged on the table, and Mrs. D. placed on her back, her hips being brought to the edge of the mattress, the thighs flexed on the pelvis, and an assistant on either side to support the feet and limbs. I then introduced the index finger of the right hand into the womb, steadying the tumor with the other hand applied to the abdomen, and succeeded in directing my finger its full length between the tumor and cervix of the uterus; this was done with great caution, for the parietes of the cervix were so extremely thin, that indiscreet manipulation would almost certainly have produced rupture of the womb. With the view, therefore, of preventing such a result, I thought it more desirable to break up the adhesions of the tumor simply with the finger than incur the hazard of introducing instruments into the uterine cavity. In proportion as the adhesions yielded, I grasped the tumor; and, without much effort, was enabled to remove it with my hands in fragments. having brought away in this manner all the solid portions of the tumor, and carrying my hand well into the cavity of the womb, I distinctly felt a sac pressing, as it were against my finger. This I immediately ruptured, and there escaped by measurement three quarts of fluid, which resembled in all its physical qualities, with the exception of the smell, pure pus. This fluid was collected in a vase as it passed from the womb, and, half an hour afterwards, on examining it, we found it no longer liquid, but presenting a solid mass, precisely like *hardened lard.* It was evident, therefore, that the temperature of the body kept this substance in a fluid state. As soon as the fluid had escaped, I introduced my hand still higher up, and felt something resembling in touch, human hair. It was, in fact, *a large mass of human hair matted together*, with no other vestige of an embryo—there was no trace of scalp, or anything else save the hair. I grasped this body, and removed it from the womb entire, it being so compact as not to separate in fragments. The womb, thus freed of its contents, contracted; and there was no loss of blood. After the solid parts of the tumor had been removed, there escaped from the bladder an incredible quantity of high-colored urine, which gave such relief to the patient, that it caused her to exclaim, in simple, yet emphatic language, "Doctor, I am in Heaven!" It may here be asked, why the catheter had not been introduced before commencing the operation. In answer, I would merely remark, that every proper attempt had been made to effect this desirable object; but it was found physically impossible, without inflicting serious injury on the patient, from the pressure of the tumor on the neck of this organ.

Mrs. D. bore the operation with a heroism which greatly surprised us; and although it became necessary to suspend occasionally all manipulation to rally her from fainting, which occurred three different times; yet, considering her extreme prostration, it may well be deemed a matter of amazement that she did not sink. The operation being completed, the patient was placed comfortably in her bed. In the course of half an hour, her breathing became easy, the pulse fell ten beats in the minute, and there was an expressure of composure about the countenance, which gave sincere joy to all of us, feeling as we did an intense and unaffected anxiety as to the immediate issue of the case. Without the aid of an anodyne, she fell into a sleep, which lasted six hours, the first repose she had enjoyed for many long nights of agony. When she awoke, she appeared greatly refreshed, and though extremely prostrated she seemed to take pleasure in gazing on her friends, to each of whom she gave a look of recognition. On the morning after the operation, her bowels were spontaneously and freely moved, a large quantity of hard fæcal matter passing away. Subsequently, simple injections of warm water sufficed to afford her a daily evacuation, and the urine was discharged freely and without obstruction. Mrs. D. continued to improve in appetitite, digestion and strength, and although her friends were admonished not to be too sanguine as to her recovery, yet they regarded the fear of any other issue as utterly groundless. On the 22d of April, fifteen days after the operation, she began to fail; and in defiance of every thing which could be brought to bear upon her case, she continued to sink, and expired on the 25th of April, having survived the operation eighteen days.

I have no doubt the anomalous mass found in the womb of this patient, was the product of a *blighted ovum*, and it may be reasonably asked whether her chances of recovery would not have been enhanced, if the tumor had been removed at an earlier period, before the powers of the system had become exhausted by long-continued and uninterrupted suffering. The adhesions, it will be remembered, of the shapeless mass to the internal surface of the womb were slight. The stearine, which escaped after the sac was punctured, I regard as nothing more than the fætal brain and other fatty portions of the system in solution. These circumstances together with the quantity of human hair removed from the womb, and the fact that the tumor was comparatively of rapid growth, are, in my judgment, strong proof of previous conception.

I cannot conclude this paper without returning my thanks to Dr. Detmold, for his prompt and efficient aid, not only during the operation, but also in the subsequent attendance. My pupils, Messrs. Burgess and Woodcock, are also entitled to the highest commendation. Throughout the case they exhibited a zeal worthy of the profession and which may be looked upon as an index of their future success.—*N. Y. Journ. of Med.*

